# Extraction of single-trial cortical beta oscillatory activities in EEG signals using empirical mode decomposition

**DOI:** 10.1186/1475-925X-9-25

**Published:** 2010-06-17

**Authors:** Chia-Lung Yeh, Hsiang-Chih Chang, Chi-Hsun Wu, Po-Lei Lee

**Affiliations:** 1 Department of Electrical Engineering, National Central University, Jhongli, Taiwan; 2 Laboratory of Integrated Brain Research, Department of Medical Research and Education, Taipei Veterans General Hospital, Taipei, Taiwan; 3 Institute of Brain Science, National Yang-Ming University, Taipei, Taiwan

## Abstract

**Background:**

Brain oscillatory activities are stochastic and non-linearly dynamic, due to their non-phase-locked nature and inter-trial variability. Non-phase-locked rhythmic signals can vary from trial-to-trial dependent upon variations in a subject's performance and state, which may be linked to fluctuations in expectation, attention, arousal, and task strategy. Therefore, a method that permits the extraction of the oscillatory signal on a single-trial basis is important for the study of subtle brain dynamics, which can be used as probes to study neurophysiology in normal brain and pathophysiology in the diseased.

**Methods:**

This paper presents an empirical mode decomposition (EMD)-based spatiotemporal approach to extract neural oscillatory activities from multi-channel electroencephalograph (EEG) data. The efficacy of this approach manifests in extracting single-trial post-movement beta activities when performing a right index-finger lifting task. In each single trial, an EEG epoch recorded at the channel of interest (CI) was first separated into a number of intrinsic mode functions (IMFs). Sensorimotor-related oscillatory activities were reconstructed from sensorimotor-related IMFs chosen by a spatial map matching process. Post-movement beta activities were acquired by band-pass filtering the sensorimotor-related oscillatory activities within a trial-specific beta band. Signal envelopes of post-movement beta activities were detected using amplitude modulation (AM) method to obtain post-movement beta event-related synchronization (PM-bERS). The maximum amplitude in the PM-bERS within the post-movement period was subtracted by the mean amplitude of the reference period to find the single-trial beta rebound (BR).

**Results:**

The results showed single-trial BRs computed by the current method were significantly higher than those obtained from conventional average method (*P *< 0.01; matched-pair Wilcoxon test). The proposed method provides high signal-to-noise ratio (SNR) through an EMD-based decomposition and reconstruction process, which enables event-related oscillatory activities to be examined on a single-trial basis.

**Conclusions:**

The EMD-based method is effective for artefact removal and extracting reliable neural features of non-phase-locked oscillatory activities in multi-channel EEG data. The high extraction rate of the proposed method enables the trial-by-trial variability of oscillatory activities can be examined, which provide a possibility for future profound study of subtle brain dynamics.

## Background

Neural network in the human brain is a dynamic system, responding to external or internal trigger events in a fraction of a second. The event-related changes in neural oscillatory activities usually contain significant physiological information, which can be either phase-locked or non-phase-locked reactive to the trigger stimuli. These oscillatory activities usually exist in specific spatial locations and in particular frequency bands [1]. Event-related power changes in oscillatory activities occur in specific frequency bands which may reflect the synchrony of certain activated neurons in the underlying neuronal population. Many clinical diagnoses report the human cortical sensorimotor rhythms, observed over the sensorimotor area and characterized by dominant frequencies in ~10 and ~20 Hz bands, as an efficacious index [2-13]. The power changes in ~20 Hz range mainly originate in the anterior bank of the central sulcus, and the ~10 Hz component is concentrated dominantly in the cortex posterior to the central sulcus. In normal subjects, voluntary movements result in a power decrease approximately 2 sec preceding movement-onsets and followed by a fast post-movement beta rebound in ~20 Hz (beta band). Previous researches have suggested that the power decrease, owes to the decrease of synchrony in the underlying neural substrate, serving as physiological meaning of motor planning and movement preparation, while the increase of power in the post-movement beta band may reflect deactivation/inhibition during the recovery phase in the movement process. Due to fast temporal changes in brain oscillatory activities, EEG (Electroencephalogrpahy) and MEG (Magnetoencephalography), with temporal resolution of a millisecond, are often chosen as powerful tools to study these oscillatory activities.

To quantify the event-induced oscillatory changes, several efficient measures have been developed for analyzing event-related oscillatory activities. Pfurtscheller et al. [6,14-16] developed an event-related desynchronization/synchronization (ERD/ERS) technique to analyze event-induced oscillatory activities of sensorimotor rhythms generated in the primary sensorimotor cortex. Clochon et al. utilized amplitude modulation (AM) method to delineate the signal envelopes of oscillatory activities [17]. Klimesch et al. further removed the rectified phase-locked components from ERD/ERS by calculating the inter-trial variance [18]. Florian and Pfurtscheller modeled the significant frequency components in oscillatory signals by means of the autoregressive (AR)-based method [19]. Salmelin et al. proposed the temporal-spectral evolution method (TSE) to filter oscillatory signals in pre-defined equi-bandwidth frequency bands followed by a rectifying and averaging process [20]. These aforementioned approaches presume a stereotypical frequency band and temporal characteristics across trials and require an average of dozens of trials for ERD/ERS calculations [21] which are unable to account for subtle trial-by-trial dynamics.

At least three techniques have been developed to extract single-trial event-related oscillatory activities. Pfurtscheller et al. utilized adaptive autoregression (AAR) and minimum Mahalanobis distance (MDA) to discriminate EEG oscillatory activities induced from four different limb movements [22,23]. Lee et al. proposed an independent component analysis (ICA)-based approach to extract post-movement beta oscillatory activities [21] and has been utilized to diagnose Parkinson's Patients [24]. Qin and He applied the Morlet wavelet to extract temporal-frequency features of movement-induced ERD/ERS while subjects performed motor imagery tasks [25]. Nevertheless, these methods extract oscillatory activities with presumed basis functions or pre-specified statistical models for signal extraction, which might be too stringent to represent single-trial information.

This paper presents an empirical mode decomposition (EMD)-based method to extract single-trial event-related oscillatory activities from EEG data. The efficacy of the proposed method has been manifested by single-trial extraction of post-movement beta ERS (PM-bERS) during self-paced right index-finger lifting task in this study. Since studies have suggested that brain oscillatory activities be stochastic and non-linearly dynamic, EMD, a powerful tool for analyzing nonlinear and nonstationary time series, might be helpful for extracting event-related oscillatory activities [19,26,27]. First, this work decomposed each EEG epoch recorded at the channel of interest (CI) into a finite number of intrinsic mode functions (IMFs) using EMD based on a sifting process [28]. Second, the spatial map of each IMF was created using the spatial weight distribution of each IMF on different EEG channels. Third, the IMFs, which retain high correlation values between their spatial maps and a pre-set spatial template, were chosen as sensorimotor-related IMFs for reconstructing noise-suppressed sensorimotor-related oscillatory activities. Finally, the reconstructed oscillatory activities were band-pass filtered within a trial-specific beta band and then rectified to detect single-trial beta rebound (BR).

This study presents an EMD-based method for extracting oscillatory activities in single-trial multi-channel EEG data. The salient feature of the proposed method is the use of a spatial map creation process to represent the spatial weights of IMFs on different EEG sensor sites, so that sensorimotor-related IMFs can be chosen by means of a spatial map matching process. The present approach requires no pre-defined statistical model or basis, which may provide a window to study intricate brain dynamics on a trial-by-trial base.

## Methods

### A. Subjects and experiments

Five healthy right-handed subjects (20-30 years old) participated in this study. The research was carried out in compliance with Helsinki declaration. All subjects gave informed consent, and the study was approved by the Ethics Committee of Institutional Review Board (IRB), Taipei Veterans General Hospital, Taiwan. All measurements were noninvasive and the subjects were free to withdraw at any time without any penalty. Subjects were asked to perform self-paced right index finger lifting approximately once every 8 seconds. Each movement was requested to be performed briskly for a duration of 200 to 300 ms, monitored by surface electromyogram (EMG) on extensor digitorum communis, with a range of finger movement around 35° ~ 40°. To prevent subjects from falling fatigue, a 5-minute break was given every 30 trials and 150 trials were acquired in each subject. EEG data were acquired by a 32-channel whole-head EEG system (band-pass, 0.05-100 Hz; sampling rate, 1000 Hz; Quick Amp., Brain Products, Co., Munich, Germany). Bipolar vertical and horizontal electrooculograms (VEOG and HEOG) were placed below and above the left eye and at the bilateral outer canthi to monitor eye movements and blinks. Epochs were segmented from EEG recordings from -4 s to 3 s anchored to movement onsets [2,20] and only those artifact-free epochs (EOG < 100 μV) were subjected to EMD decomposition.

### B. Data Analysis

#### Empirical mode decomposition of single-trial EEG epoch

**Figure 1:**
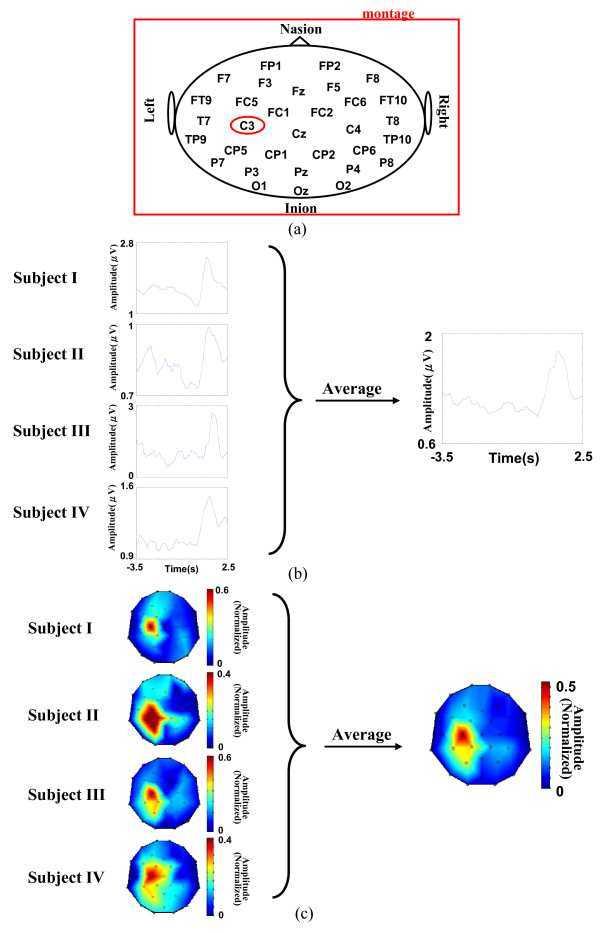
**The creation of spatial templates**. (a) The EEG montage used in this paper where C3 is marked by a red circle. (b) Conventional ERSs obtained from four subjects (template generation group) using AM method. (c) The common spatial template is created by averaging the post-movement BRs across subjects (400 trials, 100 trials for each subject, 4 subjects pooled).

Empirical mode decomposition (EMD) attempts to sequentially decompose a signal into the sum of a finite number of intrinsic mode functions (IMFs) [28-30]. Each IMF is decomposed with the following definitions: (1) the number of local extrema (including local maxima and local minima) and the number of zero-crossings must be either equal or differ at most by one, and (2) the mean value of the envelope defined by the local maxima and the envelope defined by the local minima is zero. This study manifests feasibility of the present method in extracting oscillatory activities during the right index-finger lifting task. EEG signal acquired at C3 channel (see Figure 1(a)), overlying the primary sensorimotor area (SMI) in the left hemisphere, was chosen as the channel of interest (CI) for the EMD process [14,15].

For each single movement, a single-trial EEG epoch contains *M *channels (*M* = 32) and *N *time points (*N *= 7000), arranged as an *M × N *matrix **B**. The *i*^*th *^row (*i *≤ 32) contains the measured epoch at the *i*^*th *^EEG channel, and the *j*^*th *^column in **B **contains the EEG data measured at the *j*^*th *^time point across all EEG channels. The data vector measured at CI, denoted as , is decomposed by the following EMD steps [28-30]

(1) identifying all the local extrema in , including local maxima and local minima;

(2) connecting all the local maxima/minima by a cubic spline to generate the upper/lower envelope;

(3) generating a local mean curve, , by averaging the upper and lower envelopes;

(4) calculating the pre-IMF, , by subtracting the local mean, , from , i.e., ;

(5) continuing steps (1) to (4) for *k *iterations until the difference of two continuing pre-IMFs, *SD*_*k*_, reaches a user-defined stoppage criterion, *ε *, i.e.,

where || . || denotes the Euclidean distance;

(6) setting  as the first IMF;

(7) calculating ;

(8) replacing  in step (1) by  and repeating steps (1) to (7) (sifting process), to find other IMFs, , , ⋯, and ;

(9) stopping the sifting process until the residue function  becomes a monotonic function where no more IMFs can be extracted.

**Figure 2:**
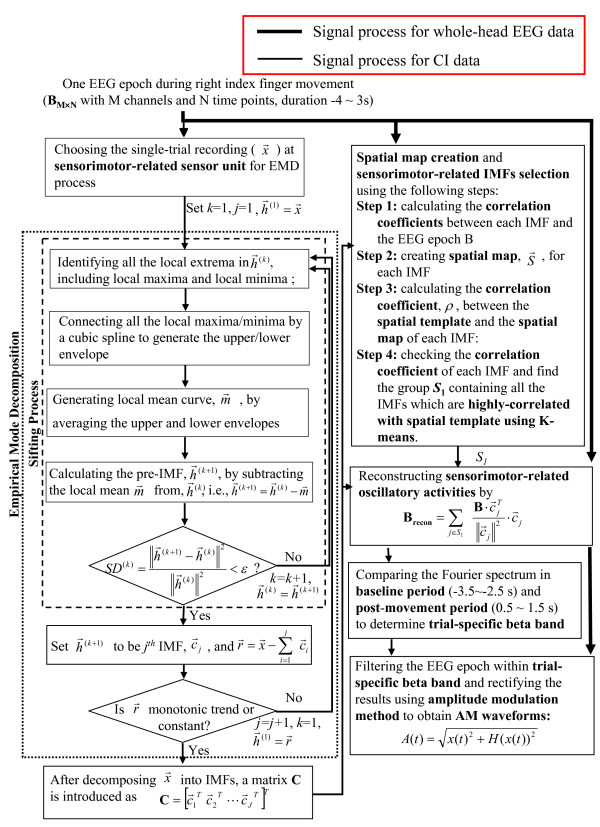
**Flow-chart of the EMD-based spatiotemporal approach**.

After applying the EMD process to a single EEG epoch (see Figure 2), the signal, *x(t)*, can be represented by a monotonic residue function, *r(t)*, plus a set of posteriori-defined IMF basis, , , ⋯, and , where *J *is the number of IMFs extracted from  and each , 1 ≤ *k ≤ J*, is a 1 × *N *vector. The IMFs can be arranged into a *J *× *N *matrix, **C**, where each row  represents the *k*^th ^IMF [11,18,19]:(1)

#### Creating a spatial map for each IMF by calculating the correlation coefficients between measured EEG data and each IMF

To obtain sensorimotor-related IMFs, this work proposes a spatial map creation process to study the spatial representation for each IMF. For each single trial, the correlation coefficients between each IMF and the EEG epoch **B **are calculated. The correlation coefficient between the epoch data at the *i*^*th *^EEG channel () and the *j*^*th *^IMF (), denoted as *p*(*i, j*), is calculated as(2)

where  and  are the mean values of  and  , respectively. The correlation coefficients between all EEG channels and the *j*^*th *^IMF are arranged into a vector , in which  ,and is designated as the spatial map for the *j*^*th *^IMF.

#### Selecting sensorimotor-related IMFs by matching the spatial map between each IMF and the spatial template using K-means

**Figure 3:**
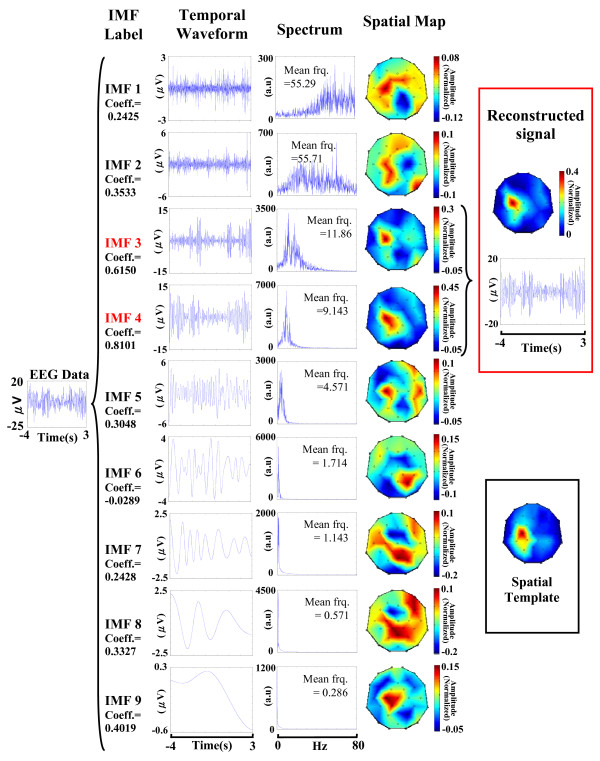
**Examples of IMF selection and signal reconstruction procedure**. IMF waveforms, Fourier spectra and spatial maps of IMF waveforms obtained from one single-trial epoch using EMD. Only IMF 3 and IMF 4, having high correlation with the spatial template, are selected for signal reconstruction.

Since unilateral index-finger movement can induce changes of oscillatory activities dominantly in the contralateral sensorimotor area [3-6], cortical activities generated from the left sensorimotor area (C3 channel) were examined in the right index-finger lifting movement study. To facilitate the selection of sensorimotor-related IMFs, we propose a spatial map matching process by correlating the spatial map of each IMF with a pre-defined spatial template. The pre-defined spatial template has focal spatial distribution over the left sensorimotor-related area, created by computing conventional ERS (see below). Those IMFs surviving high correlation-coefficient values in the spatial map matching process are then chosen for constructing noise-suppressed sensorimotor-related oscillatory activities. The correlation-coefficient value between the spatial map of the *i*^*th *^IMFs () and the spatial template () is calculated as(3)

where  and  are the mean values of  and , respectively. The correlation-coefficient values obtained between all IMFs and the spatial template are further categorized into highly-, middlely- , and lowly-correlated groups using the K-means classifier [31]. The K-means classifier determines three centriods for the three clusters by minimizing an objective function(4)

where the three clusters *S*_*j*_, *j *= *1, 2, 3 *are corresponding to highly-, middlely- , and lowly-correlated groups, and *μ*_*j *_is the centroid or mean of the all the correlation coefficients belonging to the *j*^*th *^cluster (*ρ*_*i*_∈*S*_*j*_). Only those IMFs belonging to the highly-correlated group are taken as sensorimotor-related IMFs and subjected to the subsequent reconstruction of sensorimotor-related oscillatory activities and the other IMFs are regarded as noise from bad channels. After selecting the appropriate IMFs, the sensorimotor-related oscillatory activities can be reconstructed by summating the chosen IMF portions in all EEG data matrix **B **as(5)

where ***s***_*1 *_is a group containing the index number of IMFs belonging to the highly-correlated group (see Figure 3).

#### Detecting trial-specific frequency band and extracting post-movement beta activities

**Figure 4:**
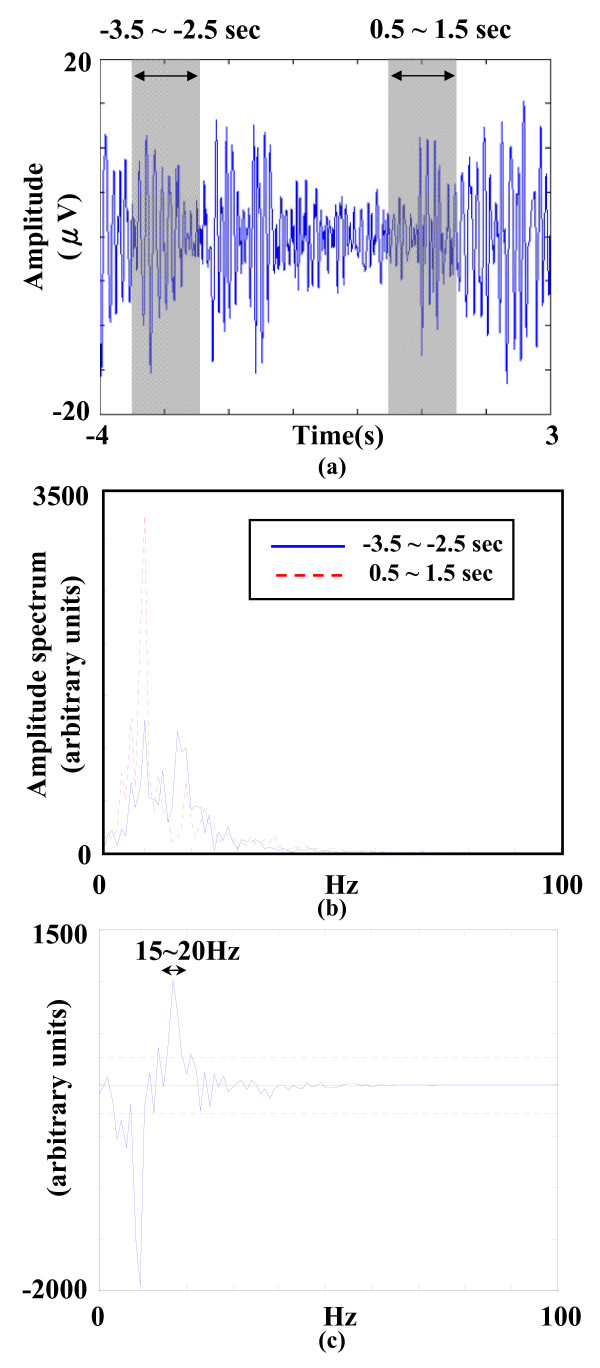
**Detecting trial-specific beta band for extracting sensorimotor-related oscillatory activities**. a) The reconstructed sensorimotor-related oscillatory activities in figure 3. (b) The trial-specific beta band was determined by comparing the post-movement Fourier spectrum (solid line) with the one estimated from baseline period (dashed line). (c) The subtracted spectrum and the trial-specific beta band (15~20 Hz).

**Figure 5:**
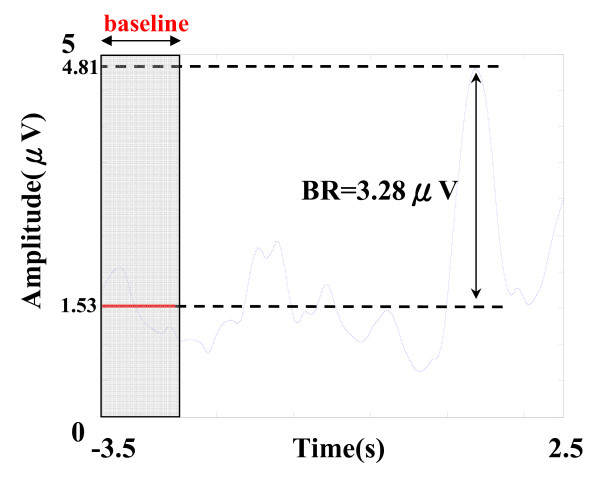
**Example of the time course of one single-trial PM-bERS in C3 channel (after filtering within 15~20 Hz) during the right index-finger lifting task**.

With the benefit of the aforementioned procedure for selecting sensorimotor-related IMFs, sensorimotor-related oscillatory activities can be well-extracted in each single trial. The trial-specific beta band is determined in each single trial to optimize the extraction of post-movement beta activities. The current work determines trial-specific beta band by comparing two Fourier spectra, one obtained from the reference period (-3.5 s ~ -2.5 s) and the other from the post-movement period (0.5 s ~ 1.5 s). The trial-specific beta band for post-movement beta activities is defined as the frequency range covering all beta-frequency components with significant modulation in terms of post-movement amplitude increase (above 95% confidence level; i.e., Z > 3.09, P < 0.01) in the differential amplitude spectrum (see Figure 4) [32]. The reconstructed sensorimotor-related oscillatory activities are band-pass filtered within the trial-specific beta band [6] to obtain *M_BP_*, and rectified by computing their AM waveforms (envelopes) using amplitude modulation (AM) method [17]. AM method detects the envelope by computing the Hilbert transform (HT) of post-movement beta activities, represented as(7)

where *M *_*BP *_(*t*) is the band-pass filtered EEG signal, *H*(*M*_*BP*_(*t*)) is its Hilbert transform and *m(t) *is the calculated AM waveform. The beta rebound (in C3 channel) is detected to evaluate the performance of the extracted post-movement beta ERS (PM-bERS). The maximum amplitude within the post-movement period (0.5 s ~ 1.5 s) in the AM waveform is then subtracted by the mean amplitude of reference period (-3.5 s ~ -2.5 s) to find the reactive activity in beta band, and only the value in the channel with maximum beta-band reactive activity is defined as beta rebound (BR) for subsequent statistics of inter-individual comparisons (see Figure 5).

#### Creating a spatial template based on conventional event-related synchronization (ERS) to facilitate the selection of sensorimotor-related IMFs

This study utilizes an electrode montage analogous to the EEG international 10¬20 system [33]. Since the EEG international 10-20 system was developed to ensure the reproducibility of EEG experiments by standardizing each EEG channel to a specified brain region, neural activities generated from a particular brain region might be able to be recognized from its spatial weight distribution over different EEG channels. This study focuses on studying the single-trial BR in the contralateral sensorimotor area. Accordingly, a spatial template, created by computing post-movement beta rebounds (post-movement BRs) in conventional event-related synchronization (ERS) over all EEG channels, was utilized to facilitate the selection of sensorimotor-related IMFs.

To demonstrate the feasibility of this standardized spatial template, four out of the five recruited participants in this study are used for generating a spatial template (template generation group), while the last participant (subject V) is used for validation (validation group). The conventional ERS technique [6,34] filters EEG data within the task-specific beta bands [6,21] and rectifies (see Figure 1(b)) the filtered signals using AM method [17] to obtain signal envelopes (see Eq. 7). The task-specific beta band is determined by the contrast between two 1-s amplitude spectra calculated over about 100 event-related EEG trials at the CI channel [6], one (serving as rest reference) is computed over the duration from -3.5 to -2.5 s preceding the onset of movement, and the other (serving as reactive target) from 0.5 to 1.5 s after the onset of movement (see Figure 4). Post-movement BR is then computed as the difference in amplitude between the maximum amplitude of ERS for each sensor site in the post-movement (0.5 to 1.5 s) interval and the mean activity between -3.5 and -2.5 s [35]. The BRs are averaged across the subjects (400 trials, 100 trials for each subject, 4 subjects pooled) to create a common spatial template (see Figure 1(c)).

## Results

Figure 3 shows an example of the EMD process in subject V. One EEG epoch (-4 s ~ 3 s) recorded at C3 position was decomposed into nine IMFs. The temporal waveforms, Fourier spectra and spatial maps of IMFs are shown in the second, third, and fourth columns, respectively. The correlation coefficients between the spatial maps of IMFs and the spatial template are 0.24, 0.35, 0.62, 0.81, 0.30, -0.03, 0.24, 0.33 and 0.40 for IMF1 to IMF9, respectively. The IMF3 and IMF4 (marked in red), with correlation coefficients categorized as the highly-correlated group (correlation coefficients are 0.62 and 0.81, respectively), were designated as sensorimotor-related IMFs for further processing. Other IMFs, which are middlely- or lowly-correlated with the spatial template, were considered as sensorimotor-unrelated IMFs and should be excluded in the following data reconstruction process to avoid deteriorating the signal-to-noise ratio. The proposed spatial map matching process provides an effective way for identifying sensorimotor-related IMFs. For example, the IMF1 and IMF2, having widely-spread spatial maps with frequencies close to 60 Hz, can be inferred as environmental electricity noise. Other IMFs (IMF5 to IMF9), whose oscillatory frequencies are low and beyond the frequency range of sensorimotor rhythms (10 Hz ~ 26 Hz [22]), might be belonged to low-frequency disturbance drifts and should be removed. Accordingly, the sensorimotor-related IMFs (IMF3 and IMF4) can be recognized by checking their spatio-temporal characteristics to reconstruct noise-suppressed sensorimotor-related oscillatory activities.

Using the aforementioned IMF selection process, sensorimotor-unrelated IMFs can be removed. The retention of high SNR in the reconstructed signals allows the detection of beta rebound (BR) could be achieved by the determination of reactive frequency band in each single trial. Figure 4(a) shows a reconstructed noise-suppressed sensorimotor-related oscillatory activity with two defined time intervals, the baseline (-3.5 s ~ -2.5 s) and post-movement (0.5 s ~ 1.5 s) periods. In Figure 4(b), the post-movement Fourier spectrum (solid line) was subtracted by the one estimated from the baseline period (dashed line) to determine the trial-specific beta band. The subtracted spectrum is shown in Figure 4(c), where the threshold indicated by the dashed lines was obtained by two times the standard deviation of the subtracted spectrum, and only those frequencies in the beta band emerging from the threshold were designated as the trial-specific beta band.

**Figure 6:**
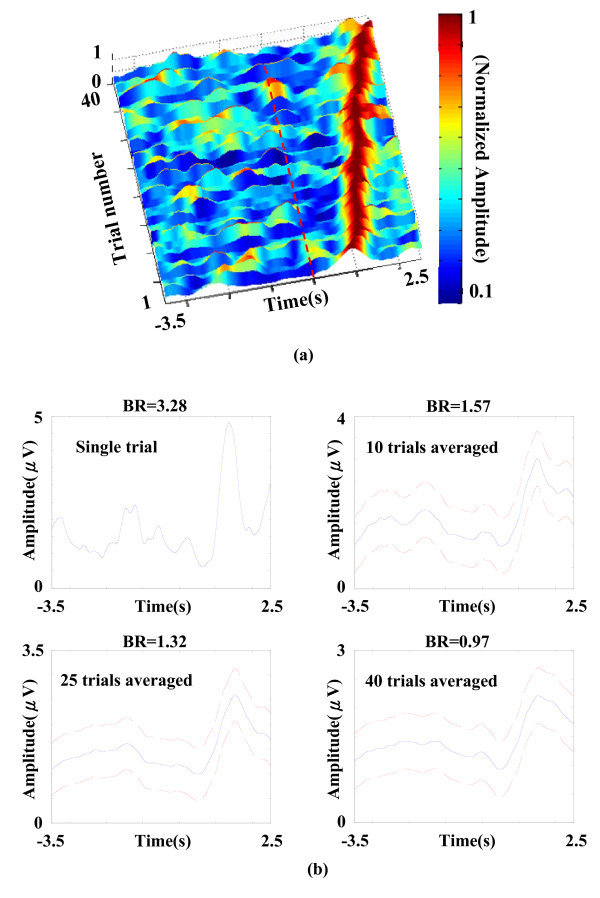
**Smearing of EEG profile and decrease of BRs due to latency jittering**. (a) The raster plot of PM-bERSs extracted from 40 single trials in subject V, where PM-bERS were sorted by the latencies of their amplitude peaks, with amplitudes normalized to their peak amplitudes. (b) The amplitudes of BRs are 3.28, 1.57, 1.32, and 0.97 μV after averaging PM-bERSs over 1, 10, 25, and 40 trials, respectively.

By filtering the sensorimotor-related oscillatory activities within the trial-specific beta band (see Figure 4(c)) and rectifying them using AM method, rectified post-movement beta ERS (PM-bERS) were obtained (see Figure 5). The peak with maximum amplitude within the post-movement period (0.5 s ~ 1.5 s) was detected and subtracted by the mean amplitude of the baseline period (-3.5 s ~ -2.5 s) to calculate the beta rebound (BR) (3.28 μv in Figure 5). Since the PM-bERS can be single-trial extracted with the help of the aforementioned IMF decomposition and data reconstruction process, the inter-trial variation in extracted PM-bERSs can be examined. Figure 6(a) shows the raster plot of PM-bERSs extracted from 40 single trials in subject V, where PM-bERSs were sorted by the latencies of their amplitude peaks, with amplitudes normalized to their peak amplitudes. The dashed line marks the timing of movement onset. Jittering was observed in the latencies of amplitude peaks in PM-bERSs across different trials (mean ± sd. = 1.64 ± 0.46 s).

Since the inter-trial latency jittering within the post-movement period varied across trials, simple averaging, such as conventional ERD/ERS methods, might attenuate and smear the resultant BR values. The amplitudes of BRs are 3.28, 1.57, 1.32, and 0.97 μV after averaging PM-bERSs over 1, 10, 25, and 40 trials, respectively (see Figure 6(b)). This demonstrates that latency jittering and inter-trial variability can inevitably lower the estimated value of beta rebound (BR).

Using the proposed EMD-based single-trial method, the current work permits BR value to be detected in a single trial which avoids the smear of BR value caused by cross-trial averaging in conventional ERS process [6,34]. The values of BRs obtained from our EMD-based method were 1.98±0.95, 1.31±0.55, 1.93±0.79, 1.87±0.44, and 1.32±0.56 μV with peak latencies of 1.64±0.46, 1.26±0.32, 1.22±0.19, 1.39 ±0.40, and 1.32 ±0.25 in the five subjects (Table 1), respectively. In contrast with BRs obtained from the conventional method, BRs amplitudes were 0.91, 0.40, 1.48, 0.53, and 0.89 μV, respectively. The single-trial BRs computed by our EMD-based method were significantly higher than the BR obtained from the conventional average method (*P *< 0.01; matched-pair Wilcoxon test). The statistical results over all the five subjects are listed in Table 1.

**Table 1:** Comparison of BRs and trial-specific beta bands between the proposed EMD-based method and the conventional filtering method

	EMD based method			Conventional filtering method		
	
Subject index	Beta rebound (*μ*V)	Trial-specific beta band (Hz)	Single-trial latency (sec)	Beta rebound (*μ*V)	Task-specific beta band (Hz)	Latency of beta rebound peak (sec)
**I**	**1.98 ± 0.95**	**17.25 ± 1.63 ~ 20.43 ± 2.60**	**1.64 ± 0.46**	**0.91**	**16.7~23.1**	**1.62**
	
**II**	**1.31 ± 0.55**	**16.62 ± 2.43 ~ 21.26 ± 2.64**	**1.26 ± 0.32**	**0.40**	**14.5~22.8**	**1.29**
	
**III**	**1.93 ± 0.79**	**16.12 ± 2.83 ~ 21.03 ± 3.00**	**1.22 ± 0.19**	**1.48**	**15.2~25.8**	**1.17**
	
**IV**	**1.87 ± 0.44**	**17.06 ± 2.70 ~ 21.96 ± 2.82**	**1.39 ± 0.40**	**0.53**	**15.4~25.2**	**1.10**
	
**V**	**1.32 ± 0.56**	**17.37 ± 2.79 ~ 20.47 ± 3.37**	**1.32 ± 0.25**	**0.89**	**15.2~23.8**	**1.22**
	
**Average**	**1.682**	**16.88 ~ 21.03**	**1.37**	**0.842**	**15.4~24.14**	**1.28**

## Discussion

Human brain rhythms are stochastic whose amplitudes, frequencies and phases can vary from trial to trial. The inter-trial fluctuations are usually contingent on a subject's performance and states, which may be linked to fluctuations in parameters of expectation, attention, arousal, and task strategy [36-41]. Therefore, those methods, such as conventional ERD/ERS techniques, using stereotypical frequency-ranges across trials for extracting neural oscillatory activities in EEG/MEG recordings, may cause single-trial reactive frequency ranges to fall out the fixed filter window [6,20] and result in misleading conclusions. This study develops an EMD-based spatiotemporal approach on EEG data to detect post-movement beta activities in a single trial. EMD is a data-driven analysis method that separates the signal into a number of IMFs without requiring prior knowledge [28]. Each IMF is an analytic and self-constructed function with time-varying frequencies. The IMF presents great flexibility to adapt itself for featuring frequency changes in a local time period [28,29]. In contrast with other conventional integration transform techniques, such as short-time Fourier transform (STFT) and wavelet-based methods, the temporal-frequency characteristics of a signal are analyzed by setting a pre-defined basis within fixed time windows, which may be too stringent to express the subtle dynamic in brain oscillatory activities. Accordingly, the proposed method may help unveil the subtle dynamics in intricate neural networks.

The proposed EMD-based method determines the trial-specific beta band for each single trial which permits extracting sensorimotor-related oscillatory activities in a single trial. Compared to conventional methods which discount subtle inter-trial changes, the task-related frequency band for band-pass filtering is fixed across all trials and the rectified oscillatory activities are averaged over a large number of trials [6]. Figure 6 shows that inter-trial jittering in latencies of amplitude peaks causes resultant BR suppression in the PM-bERS (see Figure 6(b)). Due to inter-trial variation in amplitudes, phases and oscillatory frequencies, using an ensemble average across trials is inadequate to resolve intricate neural dynamics. Table 1 demonstrates the superiority of the single-trial approach over the conventional ensemble average methods [6,34] by comparing BR values (p < 0.01).

The current proposed method is different from other EMD-based approaches [2,17,20,28,29,35,42-45]. One distinct feature is the use of spatial information (spatial map) for selecting sensorimotor-related IMF. The spatial map for each IMF is created based on the premise that any two distinct rhythmic brain signals are usually independent and uncorrelated with each other [46-49]. The distinct feature is different from other single-channel EMD studies which provide only the temporal-frequency information of IMFs. The lack of spatial information might not be comprehensive enough and could result in misleading consequences in selecting sensorimotor-related IMFs. For instance, signals outside the sensorimotor area with frequencies overlapping the beta band might be difficult to discern from temporal-frequency representations only.

Owing to the benefit of the EEG international 10-20 system, which standardizes each EEG channel to a specified brain region, researchers can render electric potentials, generated from a specific brain region, recorded by an EEG international 10-20 electrode system into a spatial map with particular spatial weight distribution. Unlike other EMD-based studies which selected sensorimotor-related IMFs by examining signal features on temporal waveforms only, we screened IMFs relevant to a specific brain region by comparing their spatial weight distribution with a customized designed spatial template. This present approach avoids the pitfall that temporal waveforms of EEG activities vary from trial-to-trial contingent upon variations in a subject's performance and state. This paper focuses on studying movement-induced beta EEG oscillatory activities in the right index-finger lifting task, and creates a spatial template with high spatial weights over the left sensorimotor area (EEG C3 channel) to facilitate selecting sensorimotor-related IMFs. Classifiers, such as K-means, artificial neural network (ANN) etc. can then automatically select sensorimotor-related IMFs. Such a combined approach is efficient for removing artifacts and extracting reliable neural features in single-trial multi-channel EEG data (see Figure 3). It is worthy to notice that this template-based IMF screening approach is simple with great flexibility. EEG activities in other brain regions can be extracted by choosing a CI for EMD computation, and designing a spatial template relevant to the interested brain region. For example, sensorimotor rhythmic activities in the right hemisphere can be extracted by designing a spatial template with spatial weight focusing on the right sensorimotor area.

## Conclusions

This paper presents an EMD-based method to extract single-trial oscillatory activities in multi-channel EEG data. The EMD-based method manifests several advantages in neurophysiological studies and clinical applications. 1) It is effective for artifact removal and extracting reliable neural features in single-trial multi-channel EEG data. 2) The method accounts for subtle trial-by-trial dynamics to preserve inter-trial variability of rhythmic activities and investigates the transitory or intermittent states in brain dynamics. 3) The single-trial approach permits an effective alternative in cases where participants cannot endure lengthy procedures or in clinical settings where patients have attention problems or are incapable of sustaining long experiments. 4) The high extraction rate of oscillatory activities could also be beneficial for brain computer interface (BCI) [6,15,50,51] which requires recognizing brain signals as control signals in few trials.

## Competing interests

The authors declare that they have no competing interests.

## Authors' contributions

CLY carried out the EEG data analysis by EMD and drafted the manuscript. HCC and CHW participated in the design of the study and performed the statistical analysis. PLL supervised the study, helped drafting and revising the manuscript. All authors read and approved the final manuscript.

## References

[B1] Jensen O Vanni S A new method to identify multiple sources of oscillatory activity from magnetoencephalographic data NeuroImage 2002 15 568 574 10.1006/nimg.2001.1020 11848699

[B2] Salmelin R Hamalainen M Kajola M Hari R Functional segregation of movement-related rhythmic activity in the human brain Neuroimage 1995 2 237 243 10.1006/nimg.1995.1031 9343608

[B3] Pfurtscheller G Neuper C Flotzinger D Pregenzer M EEG-based discrimination between imagination of right and left hand movement Electroencephalogr Clin Neurophysiol 1997 103 642 651 10.1016/S0013-4694(97)00080-1 9546492

[B4] Pfurtscheller G Pichler-Zalaudek K Ortmayr B Diez J Reisecker F Postmovement beta synchronization in patients with Parkinson's disease J Clin Neurophysiol 1998 15 243 250 10.1097/00004691-199805000-00008 9681562

[B5] Pfurtscheller G Zalaudek K Neuper C Event-related beta synchronization after wrist, finger and thumb movement Electroencephalogr Clin Neurophysiol 1998 109 154 160 10.1016/S0924-980X(97)00070-2 9741806

[B6] Pfurtscheller G Lopes da Silva FH Event-related EEG/MEG synchronization and desynchronization: basic principles Clin Neurophysiol 1999 110 1842 1857 10.1016/S1388-2457(99)00141-8 10576479

[B7] Silen T Forss N Jensen O Hari R Abnormal Reactivity of the ~20-Hz Motor Cortex Rhythm in Unverricht Lundborg Type Progressive Myoclonus Epilepsy Neuroimage 2000 12 707 712 10.1006/nimg.2000.0660 11112402

[B8] Rosell J Casanas R Scharfetter H Sensitivity maps and system requirements for magnetic induction tomography using a plannar gradiometer Physiol Meas 2001 22 121 130 10.1088/0967-3334/22/1/316 11236873

[B9] Durka PJ From wavelets to adaptive approximations: time-frequency parametrization of EEG BioMedical Engineering OnLine 2003 2 1 10.1186/1475-925X-2-1 12605721 PMC149437

[B10] Hung CI Lee PL Wu YT Chen LF Yeh TC Hsieh JC Recognition of motor imagery electroencephalography using independent component analysis and machine classifiers Ann Biomed Eng 2005 33 1053 1070 10.1007/s10439-005-5772-1 16133914

[B11] Gomarus HK Althaus M Wijers AA Minderaa RB The effects of memory load and stimulus relevance on the EEG during a visual selective memory search task: an ERP and ERD/ERS study Clin Neurophysiol 2006 117 871 884 10.1016/j.clinph.2005.12.008 16442346

[B12] Bosboom JL Stoffers D Stam CJ van Dijk BW Berendse HW Wolters ECH Resting state oscillatory brain dynamics in Parkinson's disease: an MEG study Clin Neurophysiol 2006 117 2521 2531 10.1016/j.clinph.2006.06.720 16997626

[B13] Wang ZJ Lee PWH McKeown MJ A Novel Segmentation, Mutual Information Network Framework for EEG Analysis of Motor Tasks BioMedical Engineering OnLine 2009 8 9 10.1186/1475-925X-8-9 19413908 PMC2689232

[B14] Pfurtscheller G Stancak JA Neuper C Post-movement beta synchronization. A correlate of an idling motor area? Electroencephalogr Clin Neurophysiol 1996 98 281 293 10.1016/0013-4694(95)00258-8 8641150

[B15] Pfurtscheller G Neuper C Schlogl A Lugger K Separability of EEG Signals Recorded During Right and Left Motor Imagery Using Adaptive Autoregressive Parameters IEEE transactions on Rehabilitation Engineering 1998 6 316 325 10.1109/86.712230 9749909

[B16] Pfurtscheller G Lopes da Silva FH Pfurtscheller G, Lopes da Silva FH Event-related desynchronization Handbook of Electroencephalography and Clinical Neurophysiology 1999 Elsevier Science 303 325

[B17] Clochon P Fontbonne JM Lebrun N Etevenon P A new method for quantifying EEG event-related desynchronization: amplitude envelope analysis Electroencephalogr Clin Neuro-Physiol 1996 98 126 129 10.1016/0013-4694(95)00192-1 8598172

[B18] Klimesch W Russegger H Doppelmayr M Pachinger T A method for the calculation of induced band power: implications for the significance of brain oscillation Electroencephalogr Clin Neurophysiol 1998 108 123 130 10.1016/S0168-5597(97)00078-6 9566625

[B19] Florian G Pfurtscheller G Dynamic spectral analysis of event-related EEG data Electroencephalogr Clin Neurophysiol 1995 95 393 396 10.1016/0013-4694(95)00198-8 7489668

[B20] Salmelin R Hari R Spatiotemporal characteristics of sensorimotor neuromagnetic rhythms related to thumb movement Neuroscience 1994 537 550 10.1016/0306-4522(94)90263-1 8072694

[B21] Lee PL Wu YT Chen LF Chen YS Cheng CM Yeh TC Ho LT Chang MS Hsieh JC ICA-based spatiotemporal approach for single-trial analysis of post-movement MEG beta synchronization Neuroimage 2003 20 2010 2030 10.1016/j.neuroimage.2003.07.024 14683706

[B22] Pfurtscheller G Brunner C Schlogl A Lopes da Silva FH Mu rhythm (de)synchronization and EEG single-trial classification of different motor imagery tasks Neuroimage 2006 31 153 159 10.1016/j.neuroimage.2005.12.003 16443377

[B23] Schlogl A The electroencephalogram and the adaptive autoregressive model: theory and applications Shaker Verlag 2000 Aachen

[B24] Wu CH Lee PL Wu YT Hsieh JC ICA-based analysis of movement- related modulation on beta activity of single-trial MEG measurement using spatial and temporal templates J of Medical and Biological Eng 2008 28 155 159

[B25] Qin L He B A wavelet-based time-frequency analysis approach for classification of motor imagery for brain-computer interface applications J Neural Eng 2005 2 65 72 10.1088/1741-2560/2/4/001 16317229

[B26] Dinner DS Luders H Lesser RP Morris HH Cortical generators of somatosensory evoked potentials to median nerve stimulation Neurology 1987 37 10.1212/wnl.37.7.1141 3110649

[B27] Echevarria JC Crowe JA Woolfson MS Hayes-Gill BR Application of empirical mode decomposition to heart rate variability analysis Med Biol Eng Comput 2001 39 471 479 10.1007/BF02345370 11523737

[B28] Huang NE Shen Z Long SR Wu MC Shih HH Zheng Q Yen NC Tung CC Liu HH The empirical mode decomposition and the Hilbert Spectrum for nonlinear and nonstationary time series analysis Proc Roy Soci London Ser 1998 A 903 995 10.1098/rspa.1998.0193

[B29] Huang W Shen Z Huang NE Fung YC Engineering analysis of biological variables: An example of blood pressure over 1 day Proc Natl Acad Sci USA 1998 95 4816 4821 10.1073/pnas.95.9.4816 9560185 PMC20170

[B30] Huang W Shen Z Huang NE Fung YC Nonlinear indicial response of complex nonstationary oscillations as pulmonary hypertension responding to step hypoxia Proc Natl Acad Sci USA 1999 96 1834 1839 10.1073/pnas.96.5.1834 10051555 PMC26697

[B31] Hartigan JA Wong MA A K-Means Clustering Algorithm Appl Statist 1979 28 100 108 10.2307/2346830

[B32] Pfurtscheller G Berghold A Patterns of cortical activation during planning of voluntary movement Electroencephalogr Clin Neurophysiol 1989 72 250 258 10.1016/0013-4694(89)90250-2 2465128

[B33] Lagerlund TD Sharbrough FW Jack CR Erickson BJ Strelow DC Cicora KM Busacker NE Determination of 10-20 system electrode locations using magnetic resonance image scanning with markers Electroencephalogr Clin Neurophysiol 1993 86 7 14 10.1016/0013-4694(93)90062-Z 7678393

[B34] Pfurtscheller G Aranibar A Event-related cortical desynchronization detected by power measurements of scalp EEG Electroencephalogr Clin Neurophysiol 1977 42 817 826 10.1016/0013-4694(77)90235-8 67933

[B35] Leocani L Toro C Manganotti P Zhuang P Hallett M Event-related coherence and event-related desynchronization/synchronization in the 10 Hz and 20 Hz EEG during self-paced movements Electroencephalogr Clin Neurophysiol 1997 104 199 206 10.1016/S0168-5597(96)96051-7 9186234

[B36] Earle JB Task difficulty and EEG alpha asymmetry: an amplitude and frequency analysis Neuropsychobiology 1988 20 96 112 10.1159/000118482 3253605

[B37] Hoffman RE Buchsbaum MS Escobar MD Makuch RW Nuechterlein KH Guich SM EEG coherence of prefrontal areas in normal and schizophrenic males during perceptual activation J Neuropsychiatry Clin Neurosci 1991 3 169 175 1821231 10.1176/jnp.3.2.169

[B38] Yabe H Satio F Fukushima Y Median method for detecting endogenous event-related brain potentials Electroencephalogr Clin Neurophysiol 1993 87 403 407 10.1016/0013-4694(93)90154-N 7508373

[B39] Haig AR Gordon E Rogers G Anderson J Classification of single-trial ERP sub-types: application of globally optimal vector quantization using simulated annealing Electroencephalogr Clin Neurophysiol 1995 94 288 297 10.1016/0013-4694(95)98480-V 7537201

[B40] Bastiaansen MCM Bocker KBE Cluitmans PJM Brunia CHM Event-related desynchronization related to the anticipation of a stimulus providing knowledge of results Clin Neurophysiol 1999 110 250 260 10.1016/S0013-4694(98)00122-9 10210614

[B41] Bastiaansen MCM Bocker KBE Brunia CHM Event-related desynchronization during anticipatory attention for an upcoming stimulus: a comparative EEG/MEG study Clin Neurophysiol 2001 112 393 403 10.1016/S1388-2457(00)00537-X 11165546

[B42] Huang NE Wu ML Long SR Shen SSP Qu W Gloersen P Fan KL A confidence limit for the Empirical Mode Decomposition and Hilbert spectral analysis Proc Roy Soci London Ser 2003 459 2317 2345 10.1098/rspa.2003.1123

[B43] Balocchi R Menicucci D Santarcangelo E Sebastiani L Gemignani A Ghelarducci B Varanini M Deriving the respiratory sinus arrhythmia from the heartbeat time series using empirical mode decomposition Chaos, Solitons & Fractals 2004 20 171 177

[B44] Li X Jefferys JGR Fox J Yao X Neuronal population oscillations of rat hippocampus during epileptic seizures Neural Networks 2008 21 1105 1111 10.1016/j.neunet.2008.06.002 18657392

[B45] Li X Li D Liang Z Voss LJ Sleigh JW Analysis of depth of anesthesia with Hilbert-Huang spectral entropy Clin Neurophysiol 2008 119 2465 2475 10.1016/j.clinph.2008.08.006 18812265

[B46] Stancak A Feige B Lucking CH Kristeva-Feige R Oscillatory cortical activity and movement-related potentials in proximal and distal movements Clin Neurophysiol 2000 111 636 650 10.1016/S1388-2457(99)00310-7 10727915

[B47] Babiloni C Brancucci A Babiloni F Capotosto P Carducci F Cincotti F Arendt-Nielsen L Chen AC Rossini PM Ancipatory cortical responses during the expectancy of a predictable painful stimulation. A high-resolution Electroencephalogrpahy study Eur J Neurosci 2003 18 1692 1700 10.1046/j.1460-9568.2003.02851.x 14511347

[B48] Gaetz W Cheyne D Localization of sensorimotor cortical rhythms induced by tactile stimulation using spatially filtered MEG NeuroImage 2006 30 899 908 10.1016/j.neuroimage.2005.10.009 16326116

[B49] Stavrinou ML Moraru L Cimponeriu L Della Penna S Bezerianos A Evaluation of cortical connectivity during real and imagined rhythmic finger tapping Brain Topogr 2007 19 137 145 10.1007/s10548-007-0020-7 17587169

[B50] Muller-Gerking J Pfurtscheller G Flyvbjerg H Designing Optimal spatial filters for single-trial EEG classification in a movement task Clin Neurophysiol 1999 110 787 798 10.1016/S1388-2457(98)00038-8 10400191

[B51] Babiloni C Babiloni F Carducci F Cincotti F De Pino G Del Percio C Maestrini S Priori A Tisei P Zanetti O Rossini PM Movement-related in electroencephalographic reactivity in alzheimer disease NeuroImage 2000 12 139 146 10.1006/nimg.2000.0602 10913320

